# COVID-19: Need for Equitable and Inclusive Pandemic Response Framework

**DOI:** 10.1177/0020731420967630

**Published:** 2020-10-19

**Authors:** Sabeena Mustafa, Anishia Jayadev, Maya Madhavan

**Affiliations:** 1 Department of Biostatistics and Bioinformatics, King Abdullah International Medical Research Center, Ministry of National Guard Health Affairs, Riyadh, Kingdom of Saudi Arabia; 2 Institute of Management in Government, Government of Kerala, Thiruvananthapuram, Kerala, India; 3 Department of Biochemistry, Government College for Women, Thiruvananthapuram, Kerala, India

**Keywords:** public health, mitigation, COVID19, pandemic

## Abstract

When a new infectious disease emerges as an epidemic or pandemic, strict and appropriate mitigation strategies are critical. Appropriate steps that facilitate defining of cases, carrying out accurate clinical diagnoses, and forming a powerful health surveillance that addresses public health policies and procedures are necessary. Tracking the number of COVID-19 cases over time and flattening the curve is another important element to establish research settings and identify therapeutic components to expedite and develop effective interventions. Addressing the various sections of the society in a philanthropic way is crucial to acquiring the public cooperation that is essential to controlling a disease like COVID-19. In this study, we discuss various strategies and measures adopted by Kerala, an Indian state, to combat the COVID-19 outbreak. Regular and timely updates by government public relations and health departments were used in many of the adopted strategies. The engagement of health information systems, together with the application of decentralized governance and community engagement, has contributed to effective population health management and surveillance of the pandemic.

A pandemic that has affected all sections, nationalities, and genders is ruling society with its fierce claws reaching out to the well-being of the entire world. The novel coronavirus named Severe Acute Respiratory Syndrome Corona Virus 2 (SARS-CoV-2) has claimed 1,088,515 precious lives across the world, including 110,588 in India and 742 in Kerala. The ongoing, explosive spread of SARS-CoV-2 throughout the world represents a critical challenge for all countries. COVID-19 primarily spreads through the respiratory tract, by droplets, respiratory secretions, and direct contact^1^ and is highly transmissible in humans, especially in the elderly and people with underlying diseases. Asymptomatic transmission of SARS-CoV-2 is another barrier for effective COVID-19 pandemic control because traditional public health strategies rely primarily on symptom-based disease detection to contain the spread.^2^ Currently, no therapeutic options or vaccines are available for the disease and hence prophylactic measures aimed at reducing community spread are critical. Health organizations have been working tirelessly hand in hand with governments to coordinate information flows and issue directives and guidelines to best mitigate the impact of the threat.

The first case of COVID-19 in India was an imported case from Wuhan that was reported January 30, 2020, from Kerala, a small southern state popularly known as “God’s own country.” Kerala has been effective in its strategic preparedness and response plan to fight COVID-19. This is not quite unexpected because the state had previously faced a crucial test of its substantial public health care system while combating the Nipah virus outbreak in 2018.^3^ That outbreak, characterized by the key epidemiological feature of person-to-person transmission with a high mortality rate, was successfully contained by case isolation, early initiation of barrier nursing, infection control practices, contact surveillance, and home quarantine.^4^

Kerala’s success story can be attributed primarily to 3 factors: the excellent public health care system in the state, functioning grassroot bodies, and the high level of literacy in the population. Here we discuss the strategies adopted by Kerala Government, now widely acclaimed as the Kerala Model, for mitigating the pandemic spread and executing the comprehensive preparedness at various levels of the administration for the outbreak.

## Response From the Health Care Authority About the Disease Outbreak

Ever since COVID-19 created an unprecedented social crisis touching all walks of life in Kerala, the governance mechanism could promptly discern that in a densely populated state, the casualty would be great. The Health Minister of the State immediately requested that citizens who had symptoms report or go into home quarantine. The management strategies planned and adopted by the administration conformed closely to the Global Humanitarian Response Plan launched by the United Nations on March 23, 2020, which for the global community to come to the aid of the ultra-vulnerable – people who are least able to protect themselves – which is essential for combating the viral infection.

## Steps Taken to Contain the Spread of COVID-19

Here, we summarize the existing preparedness and response strategies in Kerala to make decisions to establish a state-wide taskforce and response plan to prevent the spread of the pandemic.

### Sharing Protocols/Following Guidelines From the World Health Organization

Proper handwashing protocols as stipulated by the World Health Organization were publicized at the onset of the pandemic through various social media and audiovisual platforms. Medical experts and police department were asked to share the awareness campaign.

### Preparedness at Hospital Settings

Isolation wards were set up in hospitals, and people with doubtful health situations and contacts were kept under home quarantine. Kerala started stabilizing its health situation by implementing practices of social distancing and personal hygiene. Medical and paramedical students who had just passed their courses were appointed and trained properly to increase health care manpower as part of boosting up the machinery to meet the impending crisis. Some hospitals that had been nonfunctioning were sanitized and made ready to function.

### Initiatives and Campaigns

The government initiated a mission calling for innovative ideas and solutions from students, innovators, and startups to defend against the pandemic (see https://breakcorona.in/). Campaigns were made to familiarize the population with the new health care learnings and, for the same purpose, on March 15, the Government of Kerala introduced a new initiative (“Break the Chain”). The campaign aims to educate people about the importance of public and personal hygiene. Under this campaign, the government has installed water taps with handwash bottles at public locations, such as at the entry and exit gates of railway stations. Faculty from educational institutions ventured out to train the public and students on mass making of masks and sanitizers. All institutions and spaces such as educational institutions, cultural spaces, cinema halls, swimming pools, gymnasiums, etc., where there is a great possibility for people to come into contact, were closed. Work-from-home policies were implemented to the greatest extent possible. Mass gatherings such as marriages, festivals, religious congregations, etc., were prohibited. Strict restrictions were imposed on domestic and international travel.

### Involvement of Local Entities and Stakeholders

Civil society organizations, along with Accredited Social Health Activists (community health workers for rural Indian villages), made sure that people who were infected continued to self-isolate to prevent community spread. The Kudumbashree (Kerala State Poverty Eradication Mission), a women’s cooperative/community wing of local government, was used to address the acute shortage of hand sanitizers and face masks that occurred in many regions, and prisoners were also tasked with mask production. Citizen science initiatives^5^ and open-source public utilities came into the picture to express solidarity with the fight against the pandemic (see https://coronasafe.network/).

## Establishing Lockdown Mode

By March 23, 2020, as the count of COVID-19-positive cases in the state shot up to 91, the chief minister of the state declared a health emergency and a complete lockdown. This was indeed a bold step in the endeavor to control the situation at a very early stage. The lockdown policy implemented by Kerala was later adopted by India as a whole.

The police personnel were used to enforce lockdown instructions and drones were used to monitor mass gatherings. The intention of the state government was to test the maximum number of people by rapid test and to isolate and treat them so that conditions remained under control. For this, the government had to take care of the well-being of the entire population, irrespective of who and what they were. The government had to take into consideration the multitude of social problems that existed, such as inequality, unemployment, preexisting health issues, disability, gender issues, violence, poverty, a significant proportion of migrant laborers, etc. The various mitigation measures adopted to establish the lockdown mode and the various steps taken to make it more conducive are listed in Tables 1 and 2, respectively.

**Table 1.** Significant Mitigation Measures Adopted by the Kerala Government to Establish Lockdown Mode.

	Measure
1.	Ensured availability of medical professionals and related officials
2.	Announced an economic package of Rs. 20,000 crore
3.	Distributed social security and welfare pensions
4.	Developed mobile applications for local purchase of essentials and medicines
5.	Distributed free ration of food grains for a month for the entire population irrespective of their socioeconomic status
6.	Distributed free food materials to 1,000 transgender persons
7.	Set up and operated community kitchens with the help of local self-government authorities
8.	Made food available at a meagre rate under the Hunger-Less Kerala project
9.	Arranged for Kudumbashree workers to cook and supply food to individuals in home quarantine
10.	Arranged shelter homes and food for those who live on the streets
11.	Asked banks to consider disbursing loans at a 9% interest rate to self-help groups as a special COVID-19 package

**Table 2.** Steps Taken by the Kerala Government With a Benevolent Attitude Befitting a Welfare State to Make Lockdown Less Strenuous and More Conducive to People.

	Measure
1.	Operations in private and government offices were suspended.
2.	All international flights, both incoming and outgoing, were suspended.
3.	All means of domestic travel such as bus, taxi, and train transportation were suspended.
4.	All schools and universities were temporarily closed.
5.	Exams were cancelled for lower and upper primary classes.
6.	Exams were postponed for higher classes and colleges.
7.	Remote and online classes through virtual learning platforms were initiated.
8.	Operations of supermarkets and malls were suspended and shops selling essentials were allowed to operate for a specific time, following all the mandatory stipulations.
9.	All gatherings in parks, beaches, resorts, and public places were prohibited.
10.	Hotels/restaurants were closed temporarily except for takeaway service.
11.	Pharmacies and medical supplies remained open to serve people.

## The Use of Media in the Fight Against the Pandemic

Kerala’s success story should be credited primarily to effective dissemination of health-related information, guidelines, and awareness using both traditional and new media. Public awareness was continuously created through trolls and memes, often produced and shared by various official platforms of the state government. Various social media platforms were wisely used to convey the message with a wider reach. Preparing route maps for contact tracing of infected people was another concern, and publicizing these maps over social media proved to be highly successful. The media also started recording and sharing the experiences of people who were in isolation and quarantine and then recovered from the infection.

### Disha Helpline

A 24-hour helpline, Disha, that was set up by the Directorate of Health Services was effectively used in the management of COVID-19, providing a platform for the public to interact with and ask questions of health care experts. This was very helpful in creating awareness and removing fear and stigma among the public.

### WhatsApp Chatbot

The Health Department of the state also launched a WhatsApp Chatbot to check the spread of fake news through social media. Telemedicine facilities were also initiated by the government.

### GoK Mobile Application

A mobile application *GoK* was launched by the Government of Kerala to provide verified and authentic information from authorities directly to the general public. The information was periodically updated and made available in different languages, which made it very popular among the multilingual population.

### Digital Campaign and Press Briefings

Considering the extremely high mobile and internet penetration of the state, the creative and efficient use of digital campaigns to communicate updates about the disease to the public needs special mention. In addition, the chief minister’s regular press briefings touched on a range of issues affecting the daily lives of people during the lockdown. The approach was so compassionate that even starvation of animals was taken into consideration, when he called upon the public to feed the stray dogs and monkeys that were living on temple premises.

### Cultural Initiatives

Various cultural organizations and artists started online performances to entertain people during lockdown, helping to alleviate boredom and motivating them to engage themselves in creative ventures.

## Addressing Various Sections of Society

### Migrant Laborers

Migrant laborers, known as “guest workers,” form the major sector of the workforce that lost work and were uncertain whether to return to their respective homelands. Soon after Kerala announced the lockdown, however, the country as a whole went on a complete lockdown that prevented migrant laborers from traveling because rail and air transport were stopped. The government immediately took welfare measures to provide them with food and, in some cases, accommodation.

### Non-Resident Keralites

In the case of non-resident Keralites who live in the West and the Middle East, the situation grew alarming day by day. But the state government provided timely help to thousands of Keralites who wanted to come back to their homeland, and Norka-Roots, the official body of diaspora, provided online registration for people who wanted to return to Kerala. The state government made arrangements to test and quarantine nonresidents as they arrived at any of the 4 international airports in the state.

### Women

Women were disproportionately affected by COVID-19 because they faced several issues as soon as lockdown was implemented. These issues included domestic violence, issues related to an alcoholic spouse or head of the household, psychological issues related to fear regarding the health of themselves and family members, difficulty in facing unrest of family members who were forced to stay at home, etc. The government adopted several measures to address these issues. A 24-hour helpline number was provided to help needy women. A counseling team facilitated by district authorities catered to the various psychological needs of women in distress.

An important factor to note is the role of women in the caregiving machinery established during COVID-19. The health care system – comprising administrators, decision-makers, cleaning staff, technicians, and others – is mostly female. The same is true of personnel such as Anganwadi (rural child care center) teachers and Accredited Social Health Activists workers, not to mention the Kudumbashree members who played pivotal roles in the community kitchens where food was prepared and delivered to needy individuals and those under home quarantine.

### Elderly Individuals

Because it was known that age is a risk factor for COVID-19 as far as infectivity and associated morbidity are concerned, special attention was given from the beginning to the people of the state who are above 60 years of age, those having comorbidities, and the immunocompromised. This is particularly significant because Kerala has a large elderly population, which is predicted to grow to 8.3 million by 2026.^6^ The recovery of an elderly couple, aged 93 and 88, has brought a ray of hope to other COVID-19 patients. Some experts commented on it as the “rarest of the rare” case. This shows that recovery is possible when there is proper care.

## The Facilitating Environment

The health gains made in Kerala can be attributed to several factors, including strong emphasis from the state government on public health and primary health care, health infrastructure, decentralized governance, financial planning, girls’ education, community participation, and a willingness to improve systems in response to identified gaps – for example, in the Twelfth 5-Year Plan 2012–2017 created by the Government of India Planning Commission. Despite this investment in expanded infrastructure, by the early 1980s, there were reports of reduced access to medicine, lab supplies, and adequate sanitation (including drinking water and latrines) in public health centers in Kerala (Government of India Planning Commission. Twelfth 5-Year Plan 2012–2017. Available from: http://www.planningcommission.nic.in/plans/planrel/fiveyr/welcome.html).^7^ Thus it is apparent that more than a medical/health activity, dealing with COVID-19 is a sociopolitical activity. The strong public health system, containing a network of primary health centers, along with overall literacy have had a significant impact on the health literacy of the population. Moreover, decentralized governance gave people the ability to make decisions about their health care needs. The Navakeralam Model, which is the second phase of decentralization in Kerala, has played a significant role in refurbishing the health care system through its Ardram Mission, which aims at creating a “people-friendly” health delivery system (see https://kerala.gov.in/aardram). Through the efforts of this model, it has been proven that contagious diseases and epidemics could be contained effectively. It was the absence of such a public health model that devastated the health care system in developed nations during the pandemic.

The governance machinery, led by the chief minister and the health minister, trusted the community with efforts to contain the disease.^8^ They urged people to take care of themselves following the strict instructions given by the team dealing with COVID-19. There was rigorous monitoring of the daily activities of the community. Tests, early detection, quarantine of those who had contact with positive cases, isolation of positive cases, strict implementation of the mitigation protocol, and shielding of the vulnerable were given the utmost priority. Along with the infrastructure of the government machinery, the community also partook in prevention activities as volunteers. The majority of people who returned from abroad went into self-isolation/quarantine after screening at airports and followed government rules and regulations as part of the preventive measures. These positive attempts from the public are crucial to contain any pandemic situation and helped in Kerala’s efforts to reduce fatality rates.

## Interdepartmental Coordination

Lockdown in Kerala has made it possible to limit the number of positive cases down to less than 8,000, which would otherwise have risen up to 8 lakhs. Kerala has flattened the curve by virtue of its strategic planning, community participation, and interdepartmental coordination. Along with the network of institutions under the health department, the departments of medical education, police, fire and rescue, woman and child development, food and civil supplies, and legal metrology, together with research institutions and agencies, worked in a coordinated fashion to achieve this.

In addition to the health department, special appreciation is due for the police department. Kerala police have made effective efforts using memes and movie dialogues in the local and vernacular style to provide awareness about social distancing and washing hands. The social media page of the Kerala police, which is one of the most followed police department pages in the world with more than 1.5 million followers, was used to execute this strategy.

## Conclusion

Our results show that varied, appropriate preventive measures adopted and implemented by the Kerala state aimed to maintain strict social distancing that would be effective in reducing the magnitude of disease transmission and in delaying the peak of the disease outbreak to a certain extent. The state quickly rose to the occasion by stepping up the number of tests using the existing infrastructure and available resources. The quick and successful response should definitely be attributed to lessons learned from the Nipah experience, which can be rightly called the “dress rehearsal” for COVID-19 that shook the state in 2018 and helped the state gain confidence to handle epidemics from unfamiliar pathogens. The lessons to be learned from the community-oriented, cautiously aggressive, inclusive approach that the small state of Kerala opted for should definitely be a guidance in shaping public health policies and mitigation strategies for fighting not only this pandemic, but also infectious diseases that may arise in the future.
